# Inflammatory Response After Laparoscopic Versus Open Resection of Colorectal Liver Metastases Data From the Oslo-CoMet Trial: Erratum

**DOI:** 10.1097/01.md.0000481801.40636.7e

**Published:** 2016-03-11

In the article “Inflammatory Response After Laparoscopic Versus Open Resection of Colorectal Liver Metastases Data From the Oslo-CoMet Trial”,^1^ which appeared in Volume 94, Issue 42 of *Medicine*, the University of Oslo was not credited as the affiliation for several authors. The article has since been corrected online.
